# Urgent and Emergent Endovascular Treatment of the Downstream Aorta Soon After Open Surgical Repair in Acute Type A Aortic Dissection: Analyzing Indications and Outcomes of an Institutional Case Series

**DOI:** 10.3390/jcm15030936

**Published:** 2026-01-23

**Authors:** Peter Donndorf, Theresa Angles, Clemens Schafmayer, Justus Groß

**Affiliations:** Department of General-, Visceral-, Vascular-, Thoracic- and Transplant Surgery, University Medicine Rostock, Schillingallee 35, 18057 Rostock, Germany

**Keywords:** aortic dissection, thoracic endovascular aortic repair, malperfusion, reintervention

## Abstract

**Objectives:** Thoracic endovascular aortic repair (TEVAR) is rarely indicated on an urgent or emergent basis soon after open surgical repair of type A aortic dissection (TAAD), and systematic data on clinical outcomes are therefore missing. In the present study, we analyze a contemporary case series regarding the outcome after urgent and emergent endovascular treatment of the downstream thoracic aorta, following open surgery for TAAD. **Methods:** The study was conducted as a retrospective observational analysis. From January 2024 until April 2025, seven patients (four male, aged 56.8 ± 5.6 years) were treated with TEVAR on an urgent or emergent basis within 48 h after open surgical repair of TAAD at our institution. In all seven patients, the initial dissection extended from the ascending to the abdominal aorta. All seven patients had previously received emergent open surgical repair by ascending aortic repair combined with hemiarch replacement (five patients) or total arch replacement, utilizing the frozen elephant trunk (FET) technique (two patients). **Results:** In four patients, the indication for urgent TEVAR was due to true lumen collapse (TLC) of the downstream aorta with resulting visceral or peripheral malperfusion symptoms. Three patients were treated on an emergent basis, due to rupture of the descending thoracic aorta with a resulting hemorrhage. Technical success of the TEVAR procedure was 100%. Thirty-day mortality was 0% in the TLC cases but 66% in the ruptured cases, where two of three patients died postoperatively due to the consequences of severe hemorrhagic shock. Within the surviving patients, no subsequent aortic events occurred during follow-up. Late mortality was 0%. The follow-up period was 15.7 ± 2.0 months. **Conclusions:** In our case series, mortality of urgent or emergent TEVAR soon after open surgical repair for TAAD is substantial, especially in patients that were treated due to acute rupture of the descending thoracic aorta and consecutive hemorrhagic shock. On the other hand, true lumen collapse with resulting malperfusion was successfully treated by instant TEVAR application in all patients without late aortic complications by the midterm follow-up.

## 1. Introduction

Type A aortic dissection (TAAD) is a severe acute cardiovascular condition that requires urgent surgical intervention. Currently, a therapeutic strategy focusing on the most proximal aortic tear is mostly applied, with surgical resection of the primary entry tear in the ascending aorta and/or arch and a distal anastomosis performed open in deep hypothermic circulatory arrest, which is recommended to improve survival and promote false-lumen thrombosis [1]. The extent of surgical strategies varies from ascending aorta and hemiarch replacement to extensive resections, including the aortic arch and consecutive total arch repair, utilizing the frozen elephant trunk (FET) technique. The FET is a modification of the traditional elephant trunk technique reported by Borst and colleagues for the first time in 1983 [2], which facilitates endovascular treatment of the downstream descending aorta, if necessary, by creating a stable landing zone within the elephant trunk for later stent-graft implantation.

This is of special relevance, as 20 to 40% of the patients surviving initial TAAD develop aortic complications of the downstream aorta and frequently require further endovascular treatment [3,4].

While the results of elective endovascular distal extension through thoracic endovascular aortic repair (TEVAR), which was described for the first time by Uchida et al. [5], are good and already well described in the literature, data on urgent or emergent TEVAR following open surgical repair of TAAD are sparse.

Such data are important as borderline indications, e.g., imminent peripheral ischemia due to an insufficient expansion of the aortic true lumen can always arise following open aortic repair for TAAD, and the timing of a second aortic procedure in the vulnerable early postoperative period can be challenging.

The aim of this retrospective study was therefore to analyze a recent institutional case series, regarding the indications and outcome of early urgent or emergent TEVAR following the initial surgical procedure of the proximal aorta in patients with acute TAAD, in terms of indications for TEVAR and clinical outcomes.

## 2. Materials and Methods

### 2.1. Study Design

This was a retrospective single-center observational analysis of patients who received TEVAR on an urgent or emergent basis within 48 h after an open surgical procedure for acute TAAD from January 2024 to April 2025. During the study period, a total of 64 patients were treated surgically for TAAD at our university institution. There were 7 cases of perioperative death (10.9%), 8 cases of cerebral infarctions (12.5%) and 0 cases of paraplegia among all patients who were treated surgically for TAAD. The study complied with the Declaration of Helsinki. Due to the retrospective design of the study, no approval was required from the local ethics committee, and no informed consent was sought from patients. Patients had given general consent for their data to serve research and scientific projects.

### 2.2. Data Extraction

Data were collected retrospectively, using our center’s prospectively maintained dedicated aortic database. Demographic and baseline characteristics, intraoperative details, clinical outcomes and follow-up data were evaluated.

### 2.3. TEVAR Procedures

All patients were treated with TEVAR, utilizing the Relay Pro Thoracic stent graft system (Terumo, Tokio, Japan). TEVAR procedures were performed in a hybrid operating room under general anesthesia. Access to the common femoral artery for stent graft implantation was obtained by surgical cut-down in all cases. 

The proximal landing zone (PLZ) (i.e., native proximal descending or the stent-graft portion of a previously implanted FET) was measured and the thoracic endograft was sized accordingly, with a maximum of 10% oversizing. In all cases with a native aorta landing zone, the PLZ was located in the distal aortic arch/proximal descending thoracic aorta (Zone 3, according to the Ishimaru classification), which was distal to the origin of the left subclavian artery. In case of a PLZ in the stent-graft of a previous FET, the intended device overlap was at least 5 cm. The distal landing zone was located in the descending thoracic aorta, proximal to the origin of the celiac trunk, in all cases. Following TEVAR, all patients were managed according to the standardized postoperative protocol for endovascular aortic surgery at our intensive care unit (ICU), which includes invasive blood pressure measurement (mean arterial pressure > 80 mmHg), close neurological monitoring and hemoglobin levels above 8 mg/dL.

### 2.4. Statistical Analysis

Descriptive statistics were applied to describe the study population’s characteristics. Continuous data were documented as medians (interquartile ranges) or means (standard deviations) after controlling for normal distribution. Categorical variables were described as numbers (percentages).

## 3. Results

### 3.1. Patient Characteristics

Between January 2024 and April 2025, seven TEVAR procedures were performed after prior acute open surgical repair for the treatment of acute TAAD. The initial type A aortic dissection was classified as A E1 M0, according to the TEM classification in all seven cases. Of the patients representing this study’s cohort, four were male, and the median age at the time of TEVAR was 56.8 ± 5.6 years. The patients’ demographic characteristics and comorbidities are listed in Table 1. In all seven patients, the initial dissection extended from the ascending to the abdominal aorta.

### 3.2. TEVAR Procedure and Clinical Outcomes

All TEVAR procedures were performed within 48 h after primary open surgical repair of the ascending aorta/aortic arch on an urgent or emergent basis. Indications for TEVAR were distal true lumen collapse (TLC) of the downstream aorta with resulting visceral or peripheral malperfusion symptoms in four patients (57%), as well as rupture of the descending thoracic aorta with a resulting hemorrhage in three patients (43%). True lumen collapse (TLC) was diagnosed by CT imaging and a true lumen diameter of 1 mm or less was considered to be TLC. Malperfusion was diagnosed with clinical symptoms (pain, pulselessness, pallor) of the abdomen, lower body or lower extremities as the leading criterion. Elevated/increasing serum lactate levels were added as the secondary criterion. The median diameter of the descending aorta at the time of TEVAR was 33.2 ± 9.3 mm in all cases and 32.0 ± 3.5 mm in the ruptured cases.

**Figure 1:**
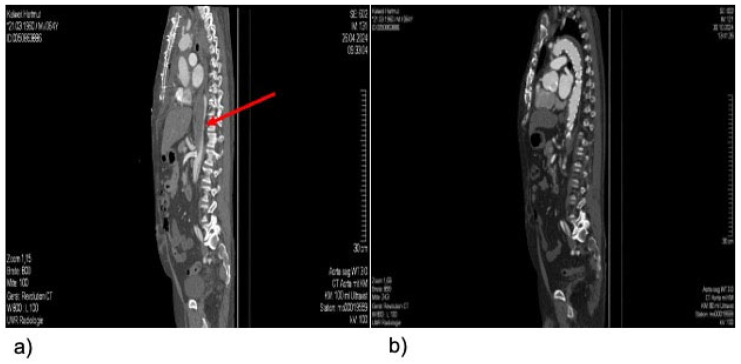
(**a**) Sagittal view from a CT examination of an exemplary patient with clinical signs of malperfusion following proximal aortic repair (FET), due to a nearly collapsed true lumen in the descending thoracic aorta (indicated by red arrow). (**b**) Sagittal view from a CT-examination of an exemplary patient (same patient as in Figure 1), demonstrating the postoperative result in the descending thoracic aorta following urgent TEVAR.

All TEVAR procedures were successfully completed, resulting in a technical success rate of 100, i.e., sealing of the torn aortic segment in the cases treated for bleeding, and sufficient expansion of the true lumen in the cases treated for true lumen collapse/malperfusion (Figure 1).

The endovascular aortic repair of the descending thoracic aorta was accomplished by utilizing two stent-grafts in three patients and one stent-graft in four patients. The intraoperative details are described in Table 2.

Thirty-day mortality was 0% in the TLC cases but 66% in the ruptured cases, where two of three patients died on postoperative day one, due to the consequences of severe hemorrhagic shock with resulting multi-organ failure. Within the surviving patients, no subsequent aortic events occurred during follow-up. Late mortality was 0% during the follow-up period of 15.7 ± 2.0 months.

## 4. Discussion

Due to its complex and dynamic nature, acute aortic dissection remains a therapeutic challenge for any cardiovascular surgeon.

In terms of acute type A aortic dissection, in the vast majority of cases, primary therapy is provided by open repair of the ascending aorta and, if necessary, the aortic arch, in order to exclude the primary entry tear, promote false lumen thrombosis and restore flow in the true lumen.

Following proximal repair in TAAD using the frozen elephant trunk technique, there was still a need for second-stage repair of the downstream aorta in around 20% [6,7] of the patients. Endovascular completion using TEVAR is safe and feasible, with high technical success rates [8]. However, second-stage surgery is usually delayed, if possible, until after the patients’ recovery from the open aortic repair, as patients are assumed to have higher mortality and morbidity in the early stage [9].

Resulting from this practice, the outcomes of elective endovascular distal extension by TEVAR are well described in the literature, but contemporary data on urgent or emergent TEVAR soon after open surgical repair of TAAD are sparse.

We present here a recent case series from our tertiary care institution, including patients undergoing urgent or emergent TEVAR within 48 h from the index open aortic repair, due to TAAD. The indication for endovascular distal extension was, in the majority of the cases, malperfusion resulting from a downstream true lumen collapse.

In general, the true lumen of the thoracic aorta should unfold after surgical proximal aortic repair, especially when performed as FET. We are not able to name the exact causes of the insufficient true lumen unfolding in the cases reported here. However, it is conceivable that persistent false lumen perfusion from more distal re-entries—although not definitely detectable in the analyzed CT scans following the prior proximal aortic repair—caused the findings reported in our series. Distal re-entries can be induced by the stent-graft portion of the FET or can occur spontaneously during the postoperative course in a diseased aortic wall.

In line with our findings, Wenkel et al. recently reported malperfusion as the dominant indication for patients undergoing early completion TEVAR within 10 days after previous FET procedures [9]. In our series, the patients treated for malperfusion had very good results: both technical and regarding the postoperative and midterm survival, which was 100%. Analyzing a series of TEVARs one month post-FET, Fortin et al. reported 0% in-hospital mortality and 100% survival during 23 months of follow-up [10]. Survival for TEVAR later than one month after open proximal aortic repair varies from 87.5 to 100% and in-hospital mortality varies from 0 to 12.5% [11,12]. Thus, it is at least encouraging that the cases treated in an urgent setting very soon after the index open surgical repair in our series match these results.

On the other hand, the patients in our series that were treated for downstream aortic rupture had a high early mortality of 66%, although this severe complication was technically solved in every case by emergent TEVAR, i.e., a sufficient seal of the ruptured aortic segment was achieved. Yet, with some of the patients still in an unstable condition, recovering from the primary proximal aortic surgery, including deep hypothermic circulatory arrest, it is conceivable that a severe complication such as aortic rupture is likely to have high mortality and morbidity. We cannot provide the exact reason for the occurrence of the early postoperative ruptures in the reported cases. Most likely, however, the false lumen was re-pressurized postoperatively due to either relatively large distal aortic new re-entry tearing or an insufficient primary entry resection during proximal surgical repair. Due to the small sample size, we are able to detect any systematic differences between the patients experiencing aortic rupture and those with postoperative malperfusion. Routine postoperative anticoagulation and blood pressure management protocols for TAAD patients were applied in the same manner in all seven cases. However, in a descriptive manner, it can be stated that all ruptured cases had previous ascending aortic and hemiarche replacement as a proximal aortic repair, where in two out of four patients treated for malperfusion, proximal aortic repair had been performed as FET.

It has been suggested by other authors that completion of TEVAR after open TAAD repair should be enhanced with candy-plug closure of the false lumen or application of the PETTICOAT or STABILISE techniques to achieve a lasting mid- and long-term remodeling of the entire aorta [13,14].

Due to the urgent or emergent clinical setting, we did not perform any enhancement techniques. A strict and multi-disciplinary follow-up protocol is therefore applied in all patients, as up to 23% of patients have been reported to require additional aortic interventions within 12 months of completion of TEVAR [8].

A relevant risk of early endovascular repair of the downstream aorta by TEVAR is the occurrence of spinal cord ischemia (SCI), especially when the FET technique is applied for proximal aortic repair. It has been demonstrated that a staging of the procedures is capable of significantly reducing the risk of this devastating complication, due to stabilized collateral networks [15].

When urgent TEVAR is indicated soon after proximal aortic repair, the use of prophylactic measures such as cerebrospinal fluid (CSF) drainage can be discussed, especially when a large aortic segment is intended to be covered. However, in our case series, we did not apply prophylactic CSF drainage and did not record any case of SCI.

Although we were able to present encouraging results for the patients in our series who were treated for malperfusion soon after open proximal aortic repair in TAAD, a delayed completion of TEVAR is certainly favorable; as early TEVAR takes place in a vulnerable period after major cardiovascular surgery and urgent endovascular surgeries with limited planning tend to take longer, patients and staff are exposed to more radiation, and patients receive more contrast agent.

However, cardiovascular surgeons might face the need for early TEVAR of the downstream aorta more often in the future, due to the trend towards a more proximal surgical aortic arch anastomosis (zone 1 or 2 instead of zone 3), which is intended to fasten and simplify the initial surgical procedure in TAAD, and towards the use of shorter stent-graft lengths in order to minimize the above mentioned risk of SCI [16]. Currently, there is growing evidence that these modifications can lead to a higher need for aortic reinterventions in general [17,18] and consistently to a higher amount of urgent or even emergent early aortic reinterventions, in particular.

Therefore, data like ours presented here—although from a small cohort, due to the rare occurrence of this specific clinical scenario—offer an empiric basis to not delay urgently indicated completion TEVAR of the downstream aorta in TAAD, even very soon after the open surgical repair of the proximal aorta.

Furthermore, our data emphasizes once more the importance of 24/7 availability of completion of TEVAR in all centers treating patients with acute type A aortic dissections.

Further knowledge on this topic, which is important for all aortic surgeons, needs to be gained, and data from randomized trials, such as the one currently recruiting, by Liu et al. [19], are awaited.

## 5. Limitations

Our study is limited by its retrospective and monocentric nature. The sample size is small. Hence, the informative value of our data is limited, as further statistical analysis for the identification of potential factors affecting the clinical outcome was not feasible.

## Figures and Tables

**Table 1:** Patients baseline characteristics. CAD: coronary artery disease; HTN: hypertension; GFR: glomerular filtration rate and SD: standard deviation.

Age (years, mean ± SD)	56.8 ± 5.6
Gender male (%)	4 (57)
HTN (%)	6 (85)
CAD (%)	1 (14)
GFR (median mL/min/1.73) (mean ± SD)	73 ± 22
Cerebrovascular event (%)	1 (14)
Diabetes mellitus (%)	2 (28)
Connective tissue disorder (%)	1 (14)
Proximal aortic repair: Hemiarch replacement (%)	5 (72)
Proximal aortic repair: FET (%)	2 (28)

**Table 2:** Intraoperative details of TEVAR procedure.

Duration of TEVAR procedure (median, min)	51 (35–90)
No. of devices (median)	1 (1–2)
Device length (median, mm)	200 (154–270)
Fluoroscopy time (median, min)	7.5 (4–21)

## Data Availability

All data are contained within the manuscript.
